# A machine learning-based predictive model for multilobar pulmonary consolidation induced by macrolide-resistant *Mycoplasma pneumoniae* pneumonia caused by the 23S rRNA A2063G mutation

**DOI:** 10.1128/spectrum.02458-25

**Published:** 2025-10-08

**Authors:** Yan Guo, Yonghan Luo

**Affiliations:** 1 Department of Reproductive Gynecology, NHC Key Laboratory of Healthy Birth and Birth Defect Prevention in Western China First People’s Hospital of Yunnan Province, Kunming, Yunnan, China; 2 Department of Reproductive Gynecology, The Affiliated Hospital of Kunming University of Science and Technology 74621, Kunming, Yunnan, China; 3 Faculty of Life Science and Technology, Kunming University of Science and Technology 47910, Kunming, Yunnan, China; 4 Second Department of Infectious Disease, Kunming Children's Hospital (Children's Hospital Affiliated to Kunming Medical University), Kunming, Yunnan, China; Children's National Hospital, George Washington University, Washington, DC, USA

**Keywords:** macrolide-resistant *Mycoplasma pneumoniae*, 23S rRNA A2063G mutation, multilobar pulmonary consolidation, machine learning, predictive model, XG-Boost

## Abstract

This study aims to develop a machine learning (ML)-based predictive model for assessing the risk of multilobar pulmonary consolidation in children with macrolide-resistant *Mycoplasma pneumoniae* pneumonia (MRMP) caused by the 23S rRNA A2063G mutation, a subgroup underrepresented in prior studies. A total of 404 MRMP cases diagnosed between October 2024 and February 2025 were included in this study. Key clinical characteristics, including laboratory test results, symptoms, and treatment outcomes, were extracted from electronic medical records. Six ML models, including Logistic Regression, Naive Bayes, K-Nearest Neighbors, Multilayer Perceptron, Random Forest, and XG-Boost, were developed to predict multilobar pulmonary consolidation. Least absolute shrinkage and selection operator (LASSO) regression was used to select relevant variables. Model performance was then evaluated using receiver operating characteristic (ROC) curves and decision curve analysis (DCA). Finally, Sharpley Additive Explanations was used for model interpretability. XG-Boost demonstrated the highest predictive performance with an area under the ROC curve of 0.976 and 0.904 in the training and validation sets, respectively, showing a high sensitivity of 0.97, specificity of 0.81, accuracy of 0.94, and an F1 score of 0.95. Key predictors identified for multilobar pulmonary consolidation included the top 10 variables: C-reactive protein, lactate dehydrogenase, fibrinogen, platelet count, albumin, hemoglobin, creatinine, aspartate aminotransferase, interleukin-6, and oxygen therapy. DCA showed that the model also exhibited strong clinical utility. The XG-Boost predictive model offers a robust tool for identifying high-risk children with MRMP caused by the 23S rRNA A2063G mutation. By integrating clinical features, the model enhances early risk stratification and can support clinical decision-making, improving the accuracy and efficiency of treatment plans.

**IMPORTANCE:**

Macrolide-resistant *Mycoplasma pneumoniae* pneumonia caused by the 23S rRNA A2063G mutation poses a significant threat to pediatric health, often leading to severe multilobar pulmonary consolidation. This study develops a high-performance machine learning model (XG-Boost) that accurately predicts this complication using key clinical indicators such as C-reactive protein, lactate dehydrogenase, and IL-6. With an area under the ROC curve of 0.976, the model enables early risk stratification, guiding clinicians in optimizing treatment for high-risk children. By improving diagnostic precision and intervention timing, this tool can reduce disease severity, minimize hospital stays, and enhance patient outcomes. The interpretability of the model via Sharpley Additive Explanations analysis further ensures its clinical applicability, making it a valuable advancement in managing antibiotic-resistant pediatric pneumonia.

## INTRODUCTION

In the post-coronavirus disease era, viral pathogens remain the predominant causes of respiratory infections in children; however, *Mycoplasma pneumoniae* (MP) continues to be an important etiological agent, particularly in lower respiratory tract infections and in older children (1–3). Studies show that *Mycoplasma pneumoniae* pneumonia (MPP) accounts for 10%–40% of community-acquired pneumonia cases in children, with a higher incidence among school-age children and adolescents (4). In recent years, the overuse of antibiotics has led to an increasing resistance of MP to macrolide antibiotics, with resistance rates in high-prevalence areas reaching over 90% (5, 6).

The resistance of MP is primarily mediated by mutations at the 23S rRNA gene locus, with the A2063G mutation being the most common resistance mechanism. The A2063G mutation has led to an increase in severe MPP cases, causing a series of pulmonary and extra-pulmonary complications (7). Pulmonary consolidation is the most common complication of MPP. Extensive consolidation not only prolongs hospitalization and increases medical costs, but it can also make the disease difficult to cure and may lead to long-term complications such as necrotizing pneumonia or obstructive bronchiolitis (8–10). Typically, the extent of consolidation correlates positively with disease severity and significantly impacts clinical treatment choices and patient prognosis.

While some studies (11) have identified risk factors for pulmonary consolidation in children with MPP, no research has focused on multilobar pulmonary consolidation associated with macrolide-resistant *Mycoplasma pneumoniae* pneumonia (MRMP) caused by the 23S rRNA A2063G mutation. Unlike previous studies (12–15) that focused on general MPP or MRMP populations, our work specifically targeted patients with a confirmed A2063G mutation, a subgroup that has been underrepresented in prior research.

Machine learning (ML) utilizes advanced algorithms and statistical techniques to process data and accurately predict disease progression, adverse outcomes, and treatment efficacy, enabling generalization and optimization (16). It has been widely applied in various aspects of medicine (17–19). However, due to the “"opaque" nature of traditional ML, the interpretability of ML models has become an increasingly important research area. Sharpley Additive Explanations (SHAP) is an interpretability algorithm based on the game-theoretic Shapley value, which ensures fairness and consistency in explanations and reveals feature interactions and their impact on the model.

Considering the above factors, this study aims to develop an ML-based predictive model to predict the risk of multilobar pulmonary consolidation in MRMP caused by the 23S rRNA A2063G mutation. By applying multiple ML algorithms, this study seeks to achieve breakthroughs in both accuracy and clinical utility, providing clinicians with an effective decision support tool.

## MATERIALS AND METHODS

### Study population

A total of 593 cases diagnosed with MPP were included from Kunming Children’s Hospital between October 2024 and February 2025.

### Inclusion criteria and exclusion criteria

#### Inclusion criteria

1. Patients under 18 years.
2. Patients meeting the clinical diagnostic criteria for MPP, in accordance with the “Guidelines for the Diagnosis and Treatment of MPP in Children (2023 Edition)” (20).

#### Exclusion criteria

1. Previous use of macrolides, tetracyclines, or fluoroquinolones before being admitted to the hospital.
2. Insufficient clinical information.
3. Past medical history of bronchopulmonary dysplasia, congenital heart disease, hematologic malignancies, inherited metabolic disorders, primary immunodeficiency, or previous use of immunosuppressive drugs.
4. Absence of the 23S rRNA A2063G mutation in MP sequencing results.

Based on the chest CT results within 2 days of hospitalization, patients were categorized into the multilobar pulmonary consolidation group if the findings indicated consolidation involving two or more lobes of the lung. All other cases were classified into the non-multilobar pulmonary consolidation group.

### Study variables and data extraction

The following clinical characteristic indicators were extracted from the hospital’s electronic medical record system: general information, symptoms and signs, laboratory tests, treatment, effect, and outcomes.

### Predictive model construction and evaluation

The positive class (multilobar pulmonary consolidation) accounted for 103/404 cases (~25%), and we relied on model-specific handling of class imbalance, rather than a specific classbalancing technique. The predictive accuracy of individual continuous variables for multilobar pulmonary consolidation was first assessed, and the correlations between them were compared, with LASSO regression used to select the optimal variables. Based on the risk factors for multilobar pulmonary consolidation, six ML models were developed using R software (version 4.4.1): Logistic Regression (LR), Naive Bayes (NB), K-Nearest Neighbors (KNN), Multilayer Perceptron (MLP), Random Forest (RF), and Extreme Gradient Boosting (XG-Boost). The data set was split into training and testing sets in a 7:3 ratio, and the predictive performance of each model was evaluated using the area under the ROC curve (AUC) in both sets. The model with the highest AUC was selected as the optimal predictive model, and its clinical applicability was further assessed using decision curve analysis (DCA). Finally, SHAP was employed to interpret and visualize the selected model.

### Statistical methods

Data were analyzed using R statistical software (version 4.4.1). For normally distributed continuous variables, data were expressed as mean ± standard deviation (*x* ± *s*), and comparisons between two groups were performed using the independent *t*-test. For non-normally distributed continuous variables, data were presented as median (interquartile range) (M [QR]), and comparisons between two groups were made using the Mann-Whitney *U* test. Categorical variables were described as frequencies and percentages (*n* [%]), and intergroup differences were assessed using the chi-square test (*χ*² test) or Fisher’s exact test, as appropriate. The significance level was set at *α* = 0.05.

## RESULTS

### Comparison of baseline characteristics between the multilobar pulmonary consolidation group and the non-multilobar pulmonary consolidation group

**Fig 1:**
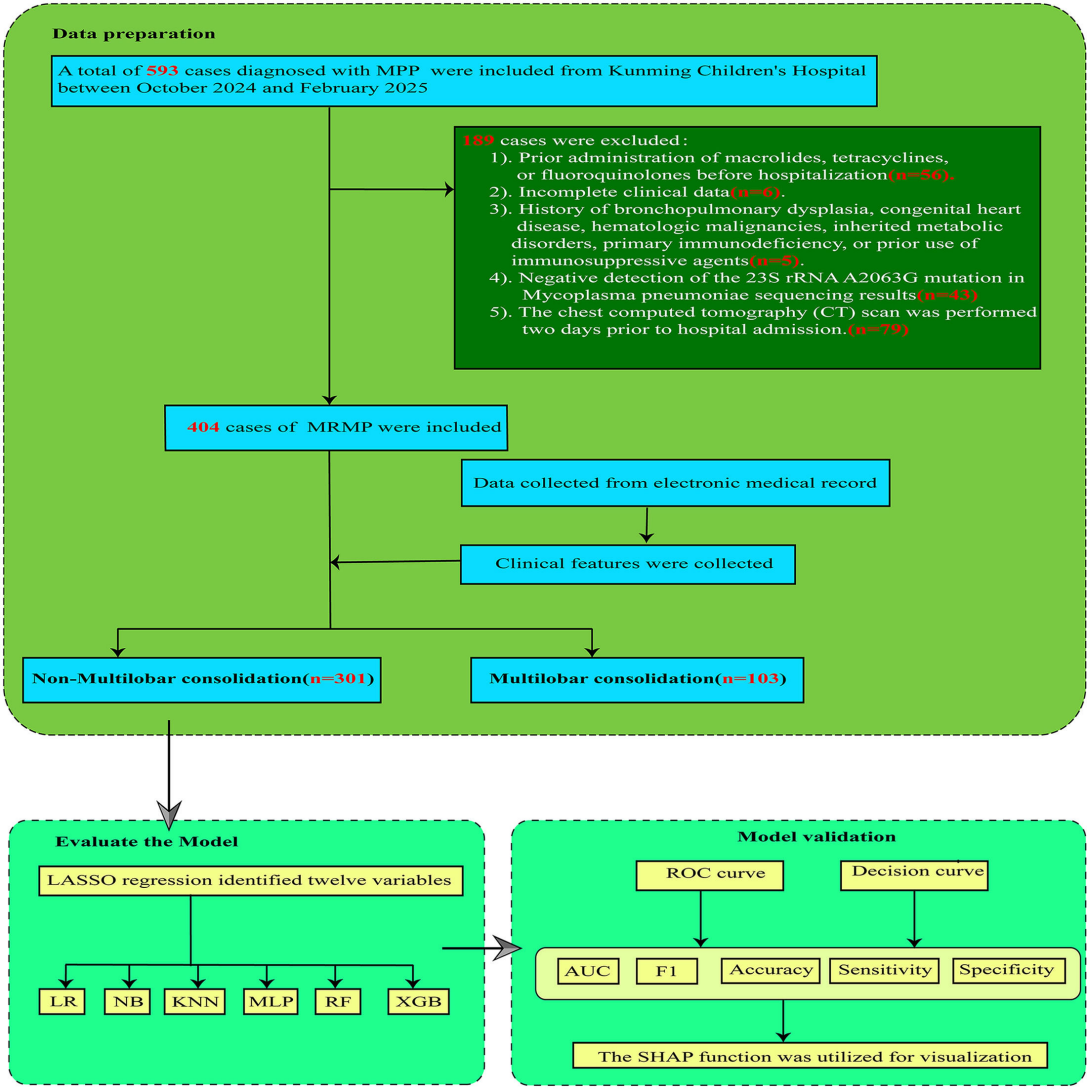
Study flowchart. LR, Logistic Regression; NB, Naive Bayes; KNN, K-Nearest Neighbors; MLP, Multilayer Perceptron; RF, Random Forest; XGB, Extreme Gradient Boosting; AUC, Area Under the ROC Curve; F1, F1-score; SHAP, Shapley Additive Explanations.

This study included 593 cases of MRMP diagnosed at Kunming Children’s Hospital between October 2024 and February 2025. Of these, 189 cases were excluded based on the following criteria: 56 cases had received macrolides, tetracyclines, or fluoroquinolones prior to hospitalization; 6 cases had incomplete clinical data; 5 cases had a history of bronchopulmonary dysplasia, congenital heart disease, hematologic malignancies, inherited metabolic disorders, primary immunodeficiency, or prior use of immunosuppressive agents; 79 cases had undergone chest CT scans prior to the 2-day admission window; and 43 cases had no detection of the 23S rRNA A2063G mutation. Ultimately, 404 MRMP cases were included in the study, and these were categorized into the multilobar pulmonary consolidation group (*n* = 103) and the non-multilobar pulmonary consolidation group (*n* = 301), as shown in Fig. 1.

Among the 404 patients with MRMP, 184 were male (46%) and 220 were female (54%). The average age of the patients was 6.23 ± 2.62 years, and the age difference between the two groups approached statistical significance (*P* = 0.05). Regarding disease duration, the overall median was 7 days, with no significant difference between groups (*P* = 0.487). The median time to diagnosis was 9 days, and there was no significant difference between the two groups in this regard (*P* = 0.724).

In terms of symptoms and signs, fever was a common symptom among all patients, with no significant difference between groups (*P* = 0.686). However, dyspnea occurred significantly more frequently in the multilobar pulmonary consolidation group compared to the non-multilobar pulmonary consolidation group (9% vs 3%, *P* = 0.018). Wheezing was more common in the multilobar pulmonary consolidation group, but the difference was not statistically significant (*P* = 0.136). Other symptoms, such as cough, decreased breath sounds, and rales, did not show significant differences between groups.

Regarding laboratory tests, there were no significant differences between the two groups in white blood cell count (WBC), neutrophil percentage (N%), or lymphocyte percentage (L%). However, absolute neutrophil count (*P* = 0.017), C-reactive protein (CRP), albumin (ALB), lactate dehydrogenase (LDH), and uric acid showed significant differences (*P* < 0.001) between the groups. Other laboratory parameters, such as hemoglobin (HGB), platelet count (PLT), and creatine kinase (CK), did not show significant differences.

Regarding treatment, 235 patients (58%) received doxycycline, with no significant difference between the groups (*P* = 0.307). Additionally, the patients received other treatments such as erythromycin (114, 28%), azithromycin (80, 20%), or fluoroquinolone antibiotics (5, 1%), with no significant differences (*P* > 0.05). A total of 252 patients (62%) were treated with cephalosporins, with no significant difference between groups (*P* = 0.129). However, the use of glucocorticoids was significantly higher in the multilobar pulmonary consolidation group (82% vs 65%, *P* = 0.002).

Regarding treatment outcomes, the length of hospital stay was significantly longer in the multilobar pulmonary consolidation group compared to the non-multilobar pulmonary consolidation group (7 days vs 6 days, *P* = 0.002). Additionally, the time to resolution of rales was significantly longer in the multilobar pulmonary consolidation group (5 days vs 4 days, *P* < 0.001). However, other treatment outcomes, such as time to recovery of body temperature, reduction of inflammatory markers, and improvement in lung imaging, did not show significant differences between the two groups (*P* > 0.05).

In terms of disease outcomes, although the multilobar pulmonary consolidation group had more severe cases (81% vs 28%, *P* < 0.001), all patients were discharged, and there were no fatalities (see Table 1).

**TABLE 1:** Comparison of clinical characteristics between the multilobar pulmonary consolidation group and the non-multilobar pulmonary consolidation group induced by macrolide-resistant *Mycoplasma pneumoniae* pneumonia caused by the 23S rRNA A2063G mutation^*a*^

Variables	Total (*n* = 404)	Non-multilobar pulmonary consolidation (*n* = 301)	Multilobar pulmonary consolidation (*n* = 103)	*P*
General information				
Sex, *n* (%)				0.434
Male	184 (46)	141 (47)	43 (42)	
Female	220 (54)	160 (53)	60 (58)	
Age, median (Q1, Q3), y	6.23 ± 2.62	6.38 ± 2.59	5.78 ± 2.67	0.05
Disease duration, median (Q1, Q3), days	7 (6, 10)	7 (6, 10)	8 (6, 10)	0.487
Time to diagnosis, median (Q1, Q3), days	9 (7, 11)	9 (7, 11)	9 (7, 12)	0.724
Symptoms and signs				
Fever, *n* (%)				0.686
NO	56 (14)	40 (13)	16 (16)	
YES	348 (86)	261 (87)	87 (84)	
Fever peak, median (Q1, Q3), °C	39.1 (38.9, 39.5)	39.1 (38.8, 39.5)	39.1 (39, 39.5)	0.108
Fever course (pre-admission), median (Q1, Q3), days	5 (3, 6)	5 (3, 6)	5 (3, 6)	0.226
Fever course (total), median (Q1, Q3), days	6 (4, 7)	6 (4, 7)	6 (3, 7)	0.261
Cough, *n* (%)				1
YES	404 (100)	301 (100)	103 (100)	
Wheezing, *n* (%)				0.136
NO	366 (91)	277 (92)	89 (86)	
YES	38 (9)	24 (8)	14 (14)	
Dyspnea, *n* (%)				0.018
NO	387 (96)	293 (97)	94 (91)	
YES	17 (4)	8 (3)	9 (9)	
Diminished breath sounds, *n* (%)				0.495
NO	340 (84)	256 (85)	84 (82)	
YES	64 (16)	45 (15)	19 (18)	
Rales, *n* (%)				0.279
NO	201 (50)	155 (51)	46 (45)	
YES	203 (50)	146 (49)	57 (55)	
Laboratory tests				
WBC, median (Q1, Q3) ,10^9^/L	7.62 (5.88, 9.84)	7.56 (5.84, 9.44)	8.13 (5.91, 11.47)	0.172
N%, median (Q1, Q3), %	59.4 (50.45, 69.05)	59 (49.9, 68.24)	62.5 (53.65, 70.4)	0.104
L%, median (Q1, Q3), %	30 (22.37, 40.1)	30.4 (22.4, 40.3)	27.9 (22.35, 39.4)	0.37
N, median (Q1, Q3) ,10^9^/L	4.67 (3.15, 6.82)	4.48 (3.09, 6.52)	5.15 (3.66, 7.06)	0.017
L, median (Q1, Q3) ,10^9^/L	2.33 (1.63, 3.2)	2.3 (1.63, 3.24)	2.41 (1.63, 3.05)	0.871
HGB, median (Q1, Q3), g/L	132 (125, 138.25)	131 (125, 138)	133 (124, 139)	0.369
CRP, median (Q1, Q3), mg/L	10.55 (3.18, 19.01)	6.58 (2.11, 14.81)	16.35 (12.37, 28.74)	<0.001
PLT, median (Q1, Q3),109/L	314.5 (254, 406)	309 (250, 400)	331 (272.5, 412)	0.093
ALT, median (Q1, Q3), U/L	12 (9, 17)	12 (9, 16)	13 (10, 19.5)	0.041
AST, median (Q1, Q3), U/L	30 (25, 37)	30 (25, 37)	30 (25, 38)	0.43
ALB, median (Q1, Q3), g/L	39.3 (37.2, 41.1)	39.9 (37.7, 41.6)	37.7 (35.05, 39.45)	<0.001
LDH, median (Q1, Q3), U/L	293 (250.88, 338)	280 (244, 315)	331 (289.75, 378)	<0.001
CK, median (Q1, Q3), U/L	70 (50.85, 103)	71 (52, 104)	69 (42, 101.55)	0.298
CK-MB, median (Q1, Q3), U/L	19 (15, 25)	19 (15, 25)	18 (14, 24.77)	0.258
BUN, median (Q1, Q3), mmol/L	3.73 (3.16, 4.43)	3.73 (3.16, 4.38)	3.78 (3.17, 4.49)	0.892
Creatinine, median (Q1, Q3), μmol/L	32 (27, 38)	32.73 (28, 38)	30 (26, 37.11)	0.049
Uric acid, median (Q1, Q3), μmol/L	258.5 (216, 308.25)	261.8 (220, 315)	239 (201, 279.5)	0.007
APTTs, median (Q1, Q3), s	36 (33.38, 39.6)	36.2 (33.2, 39.7)	35.8 (33.65, 39.55)	0.629
PT, median (Q1, Q3), s	13.4 (12.9, 14.2)	13.4 (12.9, 14.2)	13.4 (13, 14.15)	0.919
Fbg, median (Q1, Q3), g/L	3.93 (3.49, 4.37)	3.96 (3.5, 4.38)	3.92 (3.4, 4.19)	0.097
FDP, median (Q1, Q3), μg/mL	2.18 (1.47, 3.95)	2.07 (1.47, 3.95)	2.4 (1.49, 3.95)	0.476
DD, median (Q1, Q3), μg/mL	0.42 (0.24, 0.85)	0.41 (0.24, 0.85)	0.51 (0.26, 0.85)	0.052
PCT, median (Q1, Q3), ng/mL	0.25 (0.25, 0.25)	0.25 (0.25, 0.25)	0.25 (0.25, 0.25)	0.639
Ferritin, median (Q1, Q3), ng/mL	150 (106.58, 186.43)	147.9 (104.6, 186.5)	169.4 (111.2, 182.6)	0.34
IL-6, median (Q1, Q3), pg/mL	11.3 (5.36, 14.9)	10.85 (5.52, 13.71)	13.5 (5.28, 15.84)	0.395
Co-infection, *n* (%)				0.563
NO	157 (39)	114 (38)	43 (42)	
YES	247 (61)	187 (62)	60 (58)	
Treatment				
Doxycycline, *n* (%)				0.307
NO	169 (42)	121 (40)	48 (47)	
YES	235 (58)	180 (60)	55 (53)	
Erythromycin, *n* (%)				0.168
NO	290 (72)	222 (74)	68 (66)	
YES	114 (28)	79 (26)	35 (34)	
Azithromycin, *n* (%)				0.264
NO	324 (80)	237 (79)	87 (84)	
YES	80 (20)	64 (21)	16 (16)	
Quinolone antibiotics, *n* (%)				0.107
NO	399 (99)	299 (99)	100 (97)	
YES	5 (1)	2 (1)	3 (3)	
Cephalosporin, *n* (%)				0.129
NO	149 (38)	116 (39)	33 (34)	
YES	252 (62)	184 (61)	68 (66)	
Immunoglobulin, *n* (%)				0.176
NO	398 (99)	298 (99)	100 (97)	
YES	6 (1)	3 (1)	3 (3)	
Glucocorticoids, *n* (%)				0.002
NO	125 (31)	106 (35)	19 (18)	
YES	279 (69)	195 (65)	84 (82)	
Oxygen therapy, *n* (%)				<0.001
NO	334 (83)	266 (88)	68 (66)	
YES	70 (17)	35 (12)	35 (34)	
Number of bronchoscopy lavages, *n* (%)				0.21
None	226 (56)	174 (58)	52 (50)	
One	168 (42)	121 (40)	47 (46)	
Two	9 (2)	6 (2)	3 (3)	
Three	1 (0)	0 (0)	1 (1)	
Ventilator, *n* (%)				
NO	400 (99)	300 (100)	100 (97)	
YES	4 (1)	1 (0)	3 (3)	
Admission to PICU, *n* (%)				0.119
NO	396 (98)	297 (99)	99 (96)	
YES	8 (2)	4 (1)	4 (4)	
Effect				
Hospital stay, median (Q1, Q3), days	6 (5, 8)	6 (5, 7)	7 (6, 8)	0.002
Time to defervescence, median (Q1, Q3), days	1 (1, 1)	1 (1, 1)	1 (1, 1)	0.468
Time to resolution of rales, median (Q1, Q3), days	5 (4, 6)	4 (3, 5.25)	5 (4, 6.75)	<0.001
Time to decrease in inflammatory markers, median (Q1, Q3), days	5 (4, 6)	5 (4, 6)	5 (5, 7)	0.088
Time to improvement in pulmonary imaging, median (Q1, Q3), days	6 (5, 7)	6 (5, 7)	6 (5, 8)	0.91
Outcomes				
Death, *n* (%)				1
NO	404 (100)	301 (100)	103 (100)	
Severe case, *n* (%)				<0.001
NO	236 (58)	216 (72)	20 (19)	
YES	168 (42)	85 (28)	83 (81)	

^*a*^ ALT, alanine aminotransferase; AST, aspartate aminotransferase; CK-MB, creatine kinase-muscle/brain; BUN, blood urea nitrogen; APTT, activated partial thromboplastin time; Fbg, fibrinogen; FDP, fibrin degradation products; PICU, pediatric intensive care unit.

### Selection of variables

**Fig 2:**
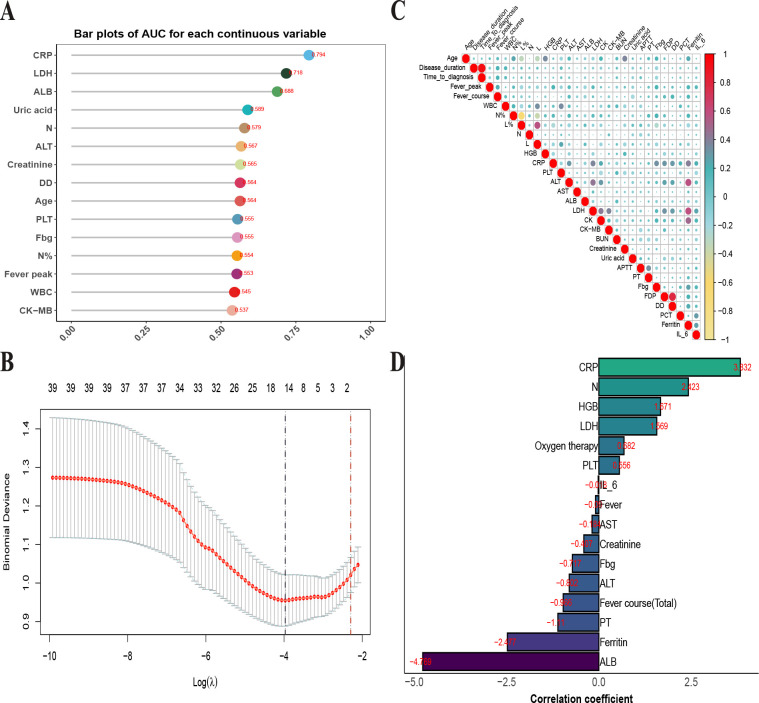
Variable selection for the predictive model by LASSO regression. (**A**) Bar chart displaying AUC values of individual continuous variables in predicting multilobar pulmonary consolidation. (**B**) Heatmap showing correlations among continuous variables; larger pie sizes indicate stronger correlations. (**C**) LASSO coefficient profiles of 39 variables plotted against the log(*λ*) sequence. The optimal penalty coefficient (*λ*) was determined using tenfold cross-validation with the minimization criterion. (**D**) Bar chart of selected variables' coefficients at lambda.min.

We attempted to construct a predictive model using relevant variables such as general information, symptoms and signs, and laboratory tests. First, univariate ROC curve analysis was conducted for all continuous variables to evaluate the predictive value of each variable for multilobar pulmonary consolidation. The results (Fig. 2A) showed that CRP, LDH, and ALB had slightly higher predictive values, but the AUC did not exceed 0.8, indicating limited practical utility. Next, we analyzed the correlations between continuous variables, revealing that some variables, such as alanine aminotransferase (ALT) and LDH, were highly correlated (Fig. 2B). To minimize the impact of multicollinearity, we employed LASSO regression for variable selection (Fig. 2C)**,** which identified 16 variables for the predictive model: CRP, N, HGB, LDH, oxygen therapy, PLT, IL-6, fever, aspartate aminotransferase (AST), creatinine, fibrinogen (Fbg), ALT, fever course (total), PT, ferritin, and ALB. The correlation coefficients of these selected variables are shown in Fig. 1D.

### Model construction and validation

**Fig 3:**
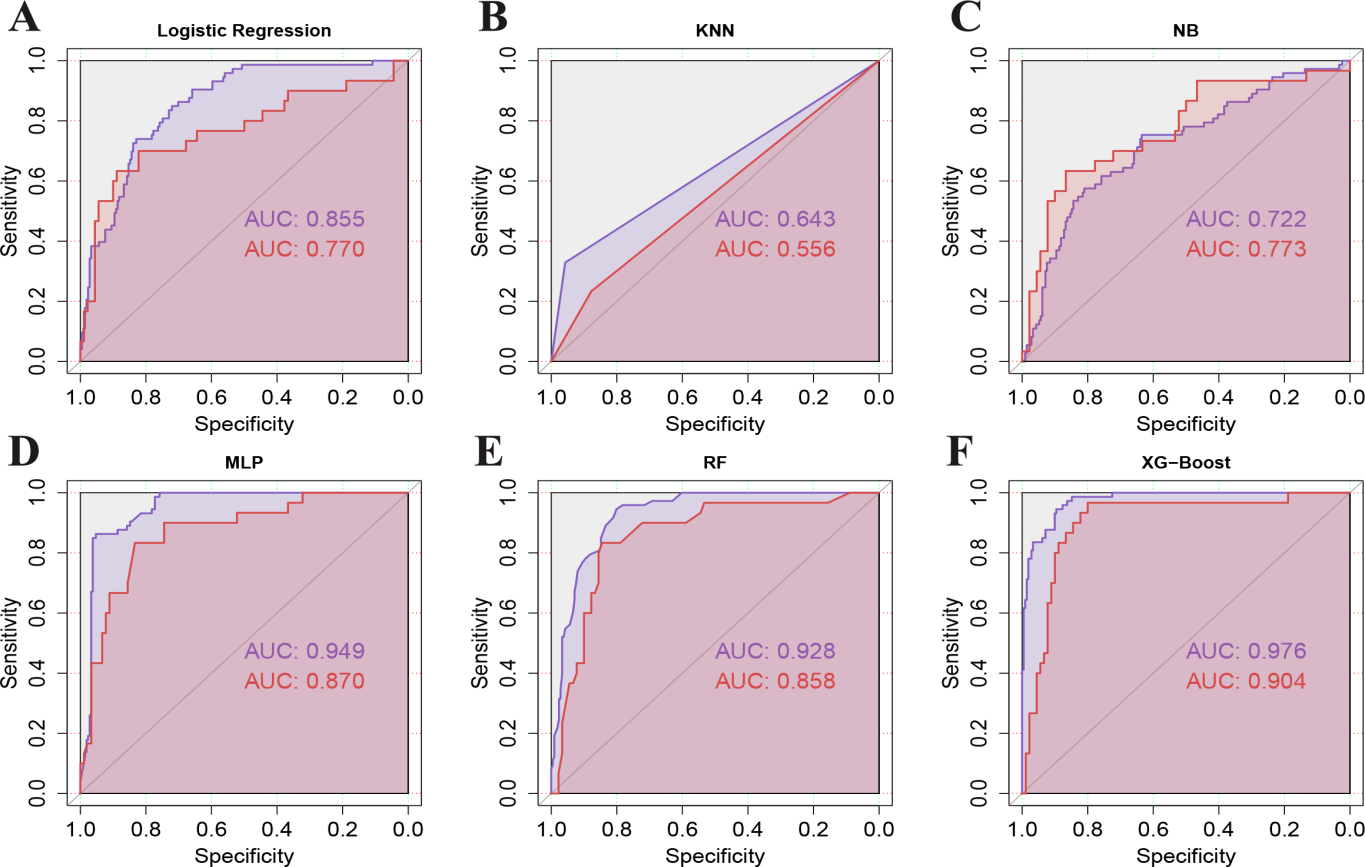
ROC curves for six machine learning models. The purple curves represent the training set, while the dark red curves represent the test set. (**A**) Logistic Regression, (**B**) k-nearest neighbors (KNN), (**C**) Naive Bayes (NB), (**D**) multilayer perceptron (MLP), (**E**) random forest (RF), (**F**) extreme gradient boosting (XGBoost).

Using the selected 16 variables, we constructed models with 70% of the total sample size for model development, and the remaining 30% was used for internal validation. We built six ML models: LR, KNN, NB, MLP, RF, and XG-Boost. The diagnostic accuracy of these six models was evaluated using ROC curves (Fig. 3), which demonstrated that XG-Boost had the highest predictive performance, with an AUC of 0.976 (95% confidence interval [CI]: 0.961–0.990) in the training set and 0.904 (95% CI: 0.838–0.969) in the validation set, showing a sensitivity of 0.97, specificity of 0.81, accuracy of 0.94, and an F1 score of 0.95.

**Fig 4:**
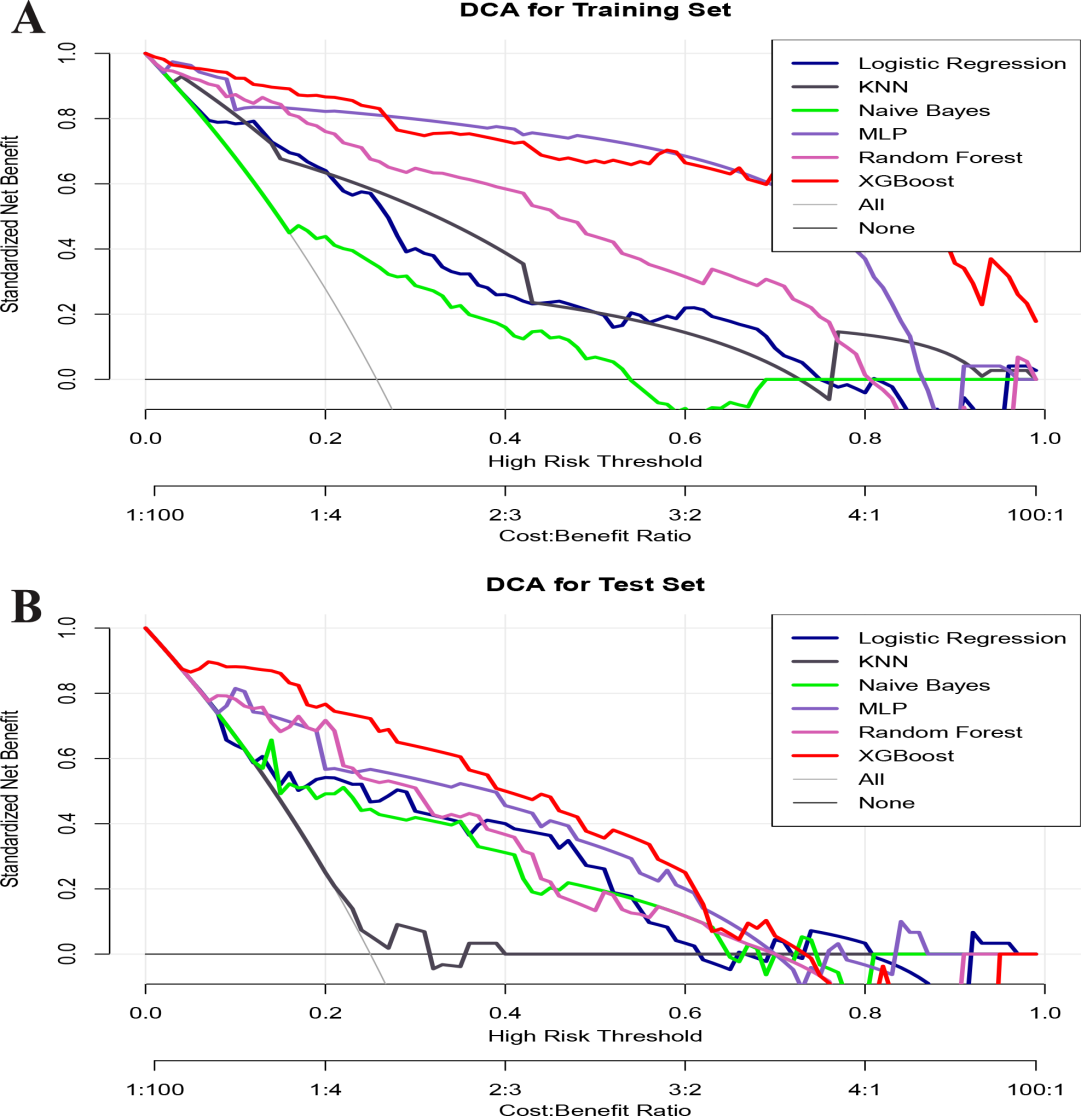
Decision curves for six machine learning models. (**A**) Training set and (**B**) test set.

**Fig 5:**
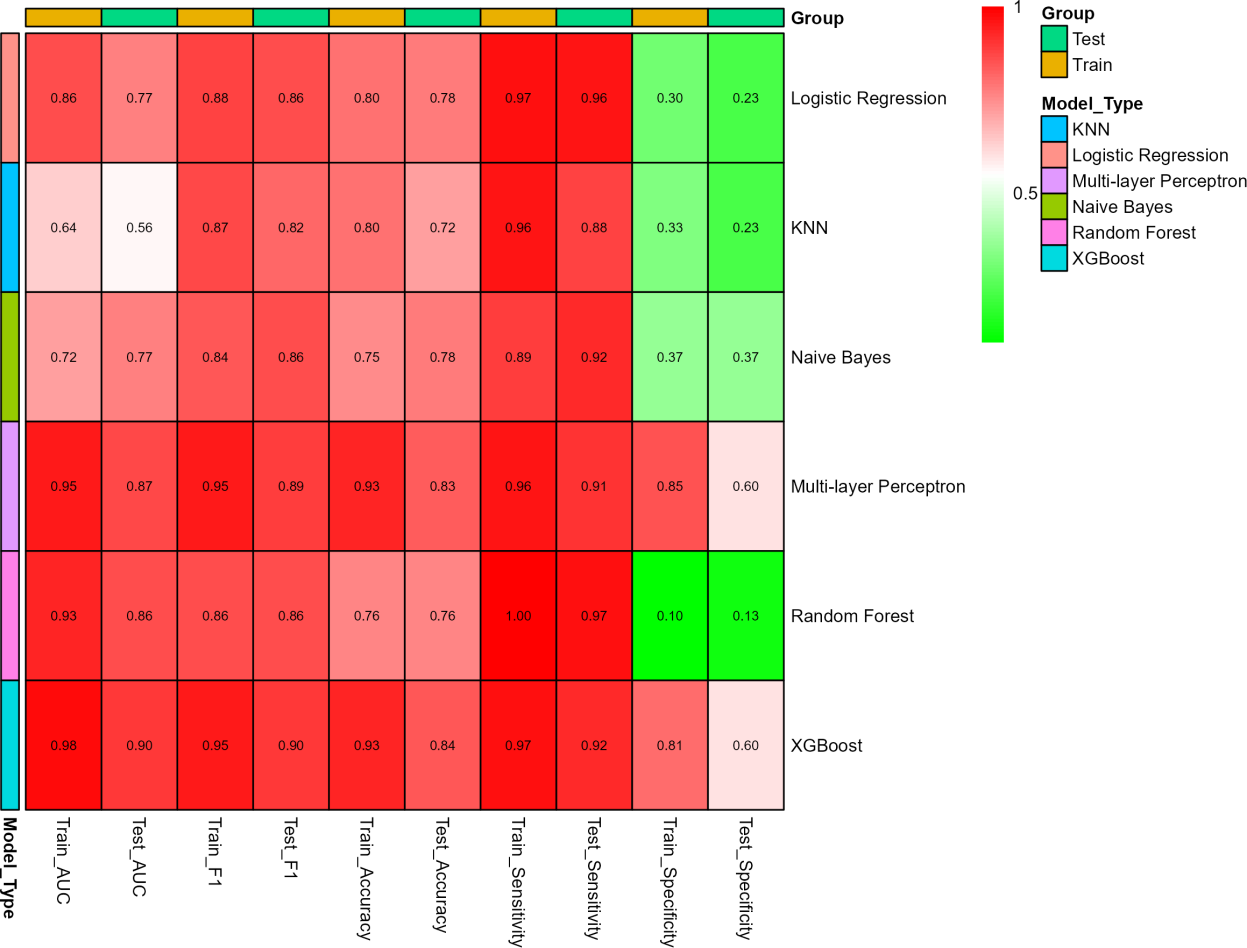
Correlation heatmap illustrating the performance metrics of six machine learning models.

Clinical applicability of the six models in both the training and validation sets was further assessed using DCA (Fig. 4), which indicated that, at high-risk thresholds (01), the XG-Boost model consistently provided positive net benefits, suggesting strong clinical utility. Additionally, a heatmap was created to display the specific performance of each model (Fig. 5).

### SHAP analysis and interpretation

**Fig 6:**
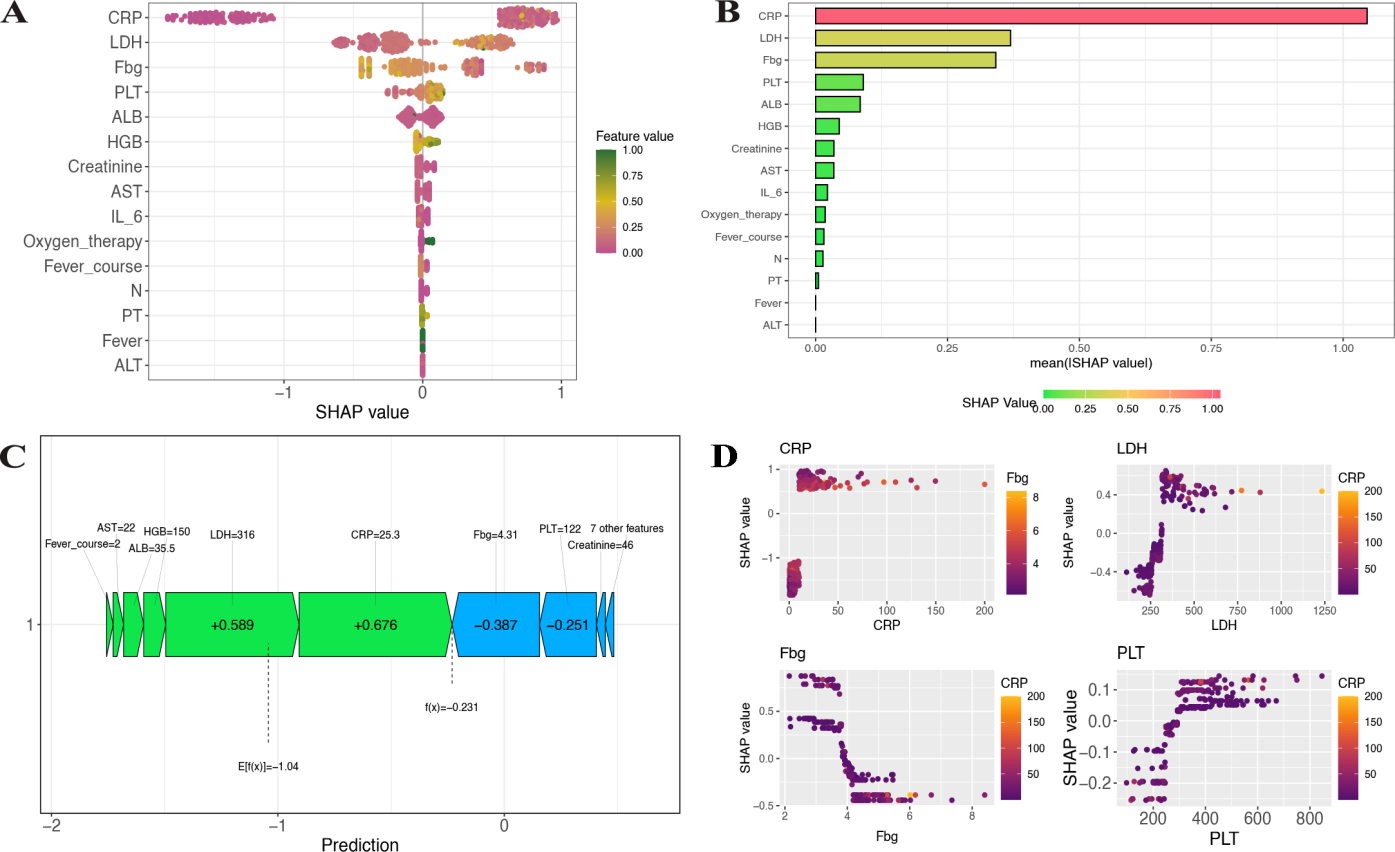
SHAP visualization of the XG-Boost model. (**A**) SHAP summary plot displaying SHAP values for all samples and features, with each row representing a feature and the *x*-axis denoting SHAP values. Green dots indicate higher feature values, while purple dots indicate lower feature values. (**B**) Comparison of mean absolute SHAP values across different variables. (**C**) SHAP force plot for multilobar pulmonary consolidation, where blue arrows indicate increased risk and green arrows indicate decreased risk. The length of the arrows represents the magnitude of impact, with longer arrows signifying a stronger influence on the prediction. (**D**) Dependency plots illustrating the relationships among the four variables with the highest SHAP values.

SHAP was utilized for visualization, ranking the SHAP values for each selected variable (Fig. 6A) and the mean SHAP values (Fig. 6B). The variables most influential to the model, ranked from highest to lowest impact, were CRP, LDH, Fbg, PLT, ALB, HGB, creatinine, AST, IL-6, oxygen therapy, fever course, N, PT, and ALT. A randomly selected patient was used for prediction, with Fig. 6C demonstrating the individual contributions of each variable to the final prediction.

Furthermore, Fig. 6D displayed the four most impactful variables, illustrating how each variable influences the SHAP values and revealing their specific effects on the predicted outcome.

## DISCUSSION

This study primarily developed an ML-based predictive model to assess the risk of multilobar pulmonary consolidation in children with MRMP caused by the 23S rRNA A2063G mutation. The main findings of the study demonstrated that, among six ML models built using 16 variables such as CRP, LDH, and Fbg, the XG-Boost model exhibited the best predictive performance, with AUCs of 0.976 and 0.904 in the training and validation sets, respectively. The model’s clinical utility was confirmed through DCA, which showed that XG-Boost provided strong clinical applicability. Additionally, SHAP visualization highlighted the importance of variables such as CRP, LDH, Fbg, and PLT in making predictions. The clinical significance of this study lies in offering a decision support tool that can be applied in clinical practice, potentially aiding in the early identification of high-risk children and optimizing treatment plans.

Macrolide resistance has become a significant global issue in recent years, particularly in the context of pneumonia caused by MP (21). The incidence of MRMP has significantly increased in East Asia. A meta-analysis (5) showed an overall resistance rate of 61% (95% CI: 54%, 68%), with notable regional differences. The resistance rates in East Asian countries, particularly in China, Japan, and South Korea, were 68% (95% CI: 63%, 73%), 61% (95% CI: 43%, 80%), and 63% (95% CI: 42%, 85%), respectively. In some reports from China, the resistance rate has reached as high as 90%, with the 23S rRNA A2063G mutation being the predominant cause (22, 23). The study conducted in Kunming, a typical region in China, represents the overall resistance levels seen in the country. According to the results of this study, the proportion of MRMP cases with the 23S rRNA A2063G mutation was found to be 90%, consistent with trends observed in both domestic and international research.

Pulmonary consolidation is one of the common complications of MPP, characterized by substantial changes in lung tissue due to the inflammatory response. The occurrence of pulmonary consolidation is typically closely related to the severity of the infection and can lead to severe complications such as necrotizing pneumonia or obstructive bronchiolitis (8–10, 24). A study by Jia et al. (11) indicated that the occurrence of pulmonary consolidation is associated with several factors, including age, duration of fever, lymphocyte count, CRP, and ferritin. Our study found that CRP is a significant factor in predicting multilobar pulmonary consolidation, which is consistent with the findings of Jia et al. and numerous other studies (25–29). Elevated CRP levels are a risk factor for severe or refractory MPP and likely play a crucial role in the pathological process as an inflammatory marker. Additionally, CRP is a well-known indicator of bacterial infections. Apart from being a systemic inflammation marker in MP infections, increased CRP should also raise concern about the potential role of concurrent bacterial infections in MRMP-induced pulmonary consolidation.

This study also found that LDH, Fbg, and ALB are important factors in predicting multilobar pulmonary consolidation. LDH, which is distributed throughout the body’s cells, serves as a marker for cell damage, and its elevation reflects the severity of pulmonary inflammation and tissue injury (30, 31). Fbg, an acute-phase reactant, may act as a marker for thrombus formation and coagulation function during lung infections, with its levels also reflecting the severity of MP infection (14, 24). ALB, on the other hand, indicates the nutritional status and immune function of the patient. Low ALB levels are commonly seen in severe cases, suggesting possible immune suppression or excessive inflammatory response. Previous studies (32) have also shown that ALB is a predictor for severe or RMPP, which is consistent with the results of this study.

In this study, all MRMP patients ultimately recovered and were discharged. Although doxycycline may pose certain risks in children under 8 years old, some studies (33–36) have confirmed that doxycycline remains an effective and relatively safe option for treating MRMP (31–34). Given the high incidence of resistant Mycoplasma, clinical practice should increasingly favor the use of doxycycline and other alternative treatment drugs. Furthermore, co-infections are common in MPP patients. In this study, approximately 60% of patients had co-infections with other bacterial pathogens, highlighting the importance of considering the risk of co-infections when treating MRMP. Additionally, 62% of patients in this study received cephalosporin antibiotics, suggesting that clinicians should pay particular attention to the management of co-infections to avoid misdiagnosis or missed diagnosis, which could affect treatment outcomes. Although LR is a commonly used and interpretable model, in this study, its AUC (0.86) was relatively low, suggesting limited discriminative capacity. In contrast, XG-Boost achieved a higher AUC of 0.98, and its strong predictive power, combined with robustness against overfitting, led us to select XG-Boost as the optimal model for predicting multilobar pulmonary consolidation in MRMP patients. In addition, a potential issue of the RF model lies in the discrepancy between its strong performance on the training set and its markedly weaker performance on the test set, particularly with respect to specificity (0.13). This indicates that the model may have overfitted the training data. Overfitting is a well-recognized challenge in ML, especially when the model is highly complex and the training data set is relatively small, as in the present study. To mitigate this issue, we considered alternative models (XG-Boost) with a lower propensity for overfitting, which demonstrated more consistent performance across both training and testing phases. Furthermore, future research should employ larger and more diverse data sets for external validation, thereby enhancing the robustness and generalizability of the model.

This study has several limitations. First, due to its retrospective design, there is inevitably a selection bias. Second, while the data set size (*n* = 404) remains relatively small for ML modeling, especially for deep models like MLP and XG-Boost. Although internal validation was performed, the lack of external validation limits the generalizability of the findings. Therefore, future research should focus on multicenter studies with larger sample sizes and prospective validation to confirm the robustness and broader applicability of the model across diverse populations.

### Conclusion

The XG-Boost predictive model offers a robust tool for identifying high-risk children with MRMP caused by the 23S rRNA A2063G mutation. By integrating clinical features, the model enhances early risk stratification and can support clinical decision-making, improving the accuracy and efficiency of treatment plans.

## Data Availability

The data sets generated and/or analyzed during the current study are not publicly available due to our research center policy, but are available from the corresponding author on reasonable request.
